# The Pathology and Treatment of Periodical Fevers

**Published:** 1847-08

**Authors:** S. G. Armor

**Affiliations:** Rockford, Illinois


ARTICLE II.

*The Pathology and Treatment of Periodical Fevers.* A paper read before the Rock River Medical Society, May 18th, 1847: By S. G. Armor, M.D., of Rockford, Illinois.

Observations on Medical Topography form a valuable feature of our Western Medical Literature. The effect of atmospheric vicissitude, of peculiar condition of soil and climate, and of the extensive decay of vegetable matter, resulting from the rich alluvial soils of the west, tend to the production of disease differing in type, in symptoms, in pathology, and consequently in treatment, from those of the same general class, as observed by emigrant physicians from the more eastern states. And it is a fact, well known to the profession, that in no forms of febrile diseases have we less extensive and carefully studied pathological observations than those of our periodical fevers of the west. Hence the necessity of the unwearied observations of those who would add to the common stock of this interesting department of our literature.

At an early period fevers were divided, arranged and systematized under various classifications according to the supposed pathology of the disease. Galen first divided into essentials and non-essentials; one a specific disease *per se;* the other sympathetic or symptomatic of the essential form. At this time the doctrines of the solidists were warmly advocated and contested. Pinel, embracing the doctrines of the humoralists, contended, with all the philosophy of the times, that the solids were only secondarily affected; the primary source of the disease being located in the fluids of the body. This was the prevailing opinion of the ancients. At a later day, Stahl published to the world, his theory of plethora, or fulness of vessels, and also a depraved condition of the fluids, fever being the *vis medicatrix natura*, an effort of nature to get rid of the onus of disease. Hoffman, maintaining the principles of solidism, taught the doctrine of derangement of the nervous system, and spasm of the capillaries. Brown, the father of the Brunonian system, also embracing the doctrines of solidism, supposed that there existed a principle of *excitability*—that a proper amount of this was necessary to the production of healthy action—too much leading to the development of fever. Broussias, enthusiastically considering that *he* had unlocked the great secret, contended that the first impression is made on the mucous membrane of the alimentary canal, and taught his pupils to look upon this as the *primum mobile* and *ultimum moriens* of ALL disease. Louis, repudiating the exclusive doctrines of all preceding theories, contends that the fluids and solids are alike affected, and labors to establish what he calls "reciprocal action" Thus, in the very onset of our investigation, we have a perfect democracy of opinion. Each, caught by the fancied beauty of his theory, has had his 'day of glory'—but has only risen to be again ushered into oblivion. The progress of physiological and pathological science is daily pointing out the errors into which, in their *exclusiveness*, these eminent authors fell; while at the same time we are enriching our medical literature by the many valuable truths which they taught.

I do not purpose, at psesent, to inquire into the character of the agency, or combination of agencies, which give rise to those forms of fever peculiar to the middle, southern and western portions of the Union. As to whether this consists in the specific poison called *malaria;* or, as has been supposed, in the influence of a "moist warm air—air excessively moist considered in connection with its own temperature and the temperature of the human body;" or on "an impaired or deranged condition of the vital functions of the skin;" does not affect my present inquiry. Each or *all* of these causes may have an agency, either directly or indirectly, in the production of our periodical fevers; and by the term *periodical* I include remittent, intermittent, and congestive fevers, which often run into each other by imperceptible gradations, and are, indeed, mere forms or modifications of the same essential disease.

The pathology of these fevers has ever been a vexed question in medicine, both as to the remote cause, and to the primary impression of that cause on the organism. But we have good reason to suppose—in fact all analogies in the pathology of disease tend to show—that the agency, be it what it may, operates primarily on the blood, by changing its normal condition. From an investigation of the great system of nutrition, as connected with animal and organic life, it must appear evident that very many disorders to which "human flesh is heir," consist in morbid alterations in the nutritive process. "Indeed," says Carpenter, in his recent work on physiology, "the progress of physiological research obviously leads to the conclusion, that in every part of the living body there is an inherent and independent vitality which enables it to grow and maintain its normal structure so long as it is supplied with the requisite materials, *and no longer;*" and the tendency which (happily for the progress of medical science) is now gaining ground, to seek in the blood for primary pathological changes, when there is an obvious general disturbance of the system, will doubtless lead to increased knowledge of the real nature of diseased states.

The next question which presents itself, is, what system is it which is principally influenced by the poison of the remote cause when mingled with the blood? That system appears to be the nervous one, and that portion of it especially, which presides over the *organic functions*, and to this pathological feature I wish particularly to direct attention.

I shall observe, for the sake of illustration, the distinction of the *cerebro-spinal* and *ganglionic* systems, so much insisted on by many physiologists, and especially by M. Bracket, in his very ingenious theory of fever. But in making the distinction, we must admit that the cerebral nerves have, to a certain extent, an influence over the organic sensibility; yet it will be at once perceived that this influence is an *indirect* one, because the viscera which perform the organic functions, do not receive their nerves from the brain, but from the ganglions. These ganglia supply the vital or nutritive functions, and have been, with propriety, considered the aggregate of so many small nervous systems, which are the peculiar centres of the organic life; just as the brain is the great and only centre of the animal life. But while the animal life immediately fails on an interruption of the organic system, the converse of the proposition does not appear to be true to the same extent. Thus fœtuses without heads, *in utero*, possess an active organic life; they are frequently, at the time of birth, monsters in bulk. Many striking and incontestible proofs might be enumerated, of the co-existence of a suspension of the cerebral functions with a permanent action of the organic life. In fact the whole process of absorption, of assimilation, of nutrition, and of the secreting operation in general is effected without any direct dependence upon the animal system. But the inquiry may next arise, as to what relation the organic nerves sustain to the general *perversion* of all these functions. Our answer is: An irritable depressed condition of organic sensibility is the immediate result of a primary pathological condition of the blood; because the system of nutrition being arrested, the natural healthy stimulus of the organic nerves is withdrawn and hence the general functional derangement characteristic of fever. It has been contended, however, by a class of pathologists, and with much apparent plausibility, that the abnormal character of the secretions is not primary and essential, but in reality a *result* of the abnormal state of the blood, from which it is separated, and that the organ itself is still performing a healthy function in separating from the blood that which would be injurious. This proposition is doubtless too extensive and too broad a one. It is easy to conceive, and indeed rational to suppose that derangement of *healthy organic sensibitity* is equally concerned in the production of faulty functional derangement of the secernant system; thus beautifully illustrating the doctrines of "reciprocal action" as taught by Louis. And to this law of relation, with the curative deductions therefrom, our attention should be particularly directed.

All the symptomatology of our periodical fevers, points to an adynamic condition of the nervous system as the most prominent and formidable lesion. Indeed, the very fact of their *periodical* character throws much light on their pathology, for periodicity is characteristic of diseases of the nervous system. In the examination of a patient, we observe a dry, hot skin, increase of vascular excitement, and general functional derangement of all the secernant system. But these symptoms, it will be borne in mind, are secondary; they have been announced by chill, which is occasioned by impression made on the nervous system. We are led to the conclusion, therefore, that fever, as a *symptom*, is merely reaction from nervous prostration. Break down, from any cause, the nice balance existing between the nervous and vascular systems, and fever always follows. Take, for example, concussion of the brain. First, we have entire prostration of nervous energy, body cold and chilly, secretions measurably suspended; reaction eventually comes on, and fever, thirst, hot skin, dry mouth, in short all the symptoms of fever are present.

Post mortem examinations have frequently pointed out extensive structural lesions in these forms of fever; but the existence of these lesions by no means determined the true and original character of the disease; for the fever may run through its entire course *without* their existence. But if the paroxysms be not promply arrested, it is not strange that local inflammatory action should become speedily developed during the progress of the fever. The harmony of function, and the equal distribution of nervous and vascular excitement are entirely broken up. One organ may be excited, another depressed; the heart and arteries excited, and the muscular system perhaps languid; increased activity of the intestinal exhalents, and diminished activity of the exhalents of the surface; copious secretions from the kidneys, and torpor of the liver. Thus, the proper balance is broken up, the result of which is an excited condition of the entire organism, and frequently the production of local inflammations.

In relation to functional lesions, the liver is found very uniformly complicated; giving, in consequence of the marked disorders of the biliary secretions usually present, the almost universal name of *bilious* to our remittent fevers of the west — a name highly objectionable, if we are to understand thereby that the fever depends essentially upon disease of the liver, or upon vitiated bile as its proximate cause. The same objection applies to the names cephalitic, gastric, and enteric remittents, for they point out local lesions which are but accidents and consequences of successive paroxysms of congestion and reaction; yet they are unhappily, too frequently mistaken for the original basis of the disease.

Another frequent and often fatal complication, is inflammation of the mucous membrane of the gastro-intestinal canal. Drs. Gerhard and Stewardson have recorded a number of cases which occurred in the Pennsylvania Hospital in the year 1834, and again in the years 1838, 1839, and 1840, in which this lesion was uniformly present; sometimes resulting in melonation, and changes in thickness, consistance, and color, variously combined in different cases; yet in no case was there disease of the elliptical plates, or mesenteric glands, or glands of Peyer and Brunner, occurring in the typhoid fevers of the New England States. It is not strange, however, that gastro-enteric inflammation becomes so frequently a complication in our periodical fevers, for, reasoning *a priori*, and from anatomical structure, this lesion would become readily developed as a secondary pathological condition during the progress of the fever. And the fact should never be lost sight of, that this local phlegmasia is increased by each successive paroxysm. These local inflammations grow out of the pathological condition to which I have alluded. In the first place congestion, or the cold stage, is merely imperfect reaction, and reaction always supposes primary prostration. This broken down condition of nervous energy results from the virulence of the remote cause. The functions of the capillaries being also morbidly impressed, the blood is consequently thrown into the centre of the circulation; and the heart, dependent for its action on nervous energy received from the brain, becomes enfeebled in its action, and the result is engorgement of blood in the portal circle· Having no valves, the hepatic veins and their radicals dispersed through the liver become engorged; the *vena partorum* and its radicals also become congested; then the abdominal viscera fall into a state of congestion; all this resulting from the want of valvular structure and from debility of the right side of the heart. Protracted venous congestion, by the excitement of distension may become the cause of the active variety. Hence disease of the entire portal circle constitutes the essential anatomical characteristic of our remittent and intermittent fevers.

Finally, as corroborative evidence of the pathological views here presented, an inference may be drawn from the remedies successfully employed in their cure. In intermittent diseases purely neuralgic in character, we resort to those remedies which are anti-periodic and sedative in their effects; and does it not throw light on the true character of periodical fevers that the same remedies, when properly administered, have the like effect in breaking up the chain of morbid action? By the concurrent and almost unanimous testimony of the profession, the preparations of cinchona, variously combined with anodynes, are found to be the most specific in arresting both forms of disease; and it is yet an interesting inquiry as to whether we may not find in other remedies, whose influence is exclusively and almost specifically directed to the nervous system, powerful remedial agents in the cure of intermittent fevers.

If the true theory of periodical fever be such as I have represented it, the indications to be accomplished in their treatment are mainly two. First, to prevent the recurrence of the paroxysms; second, to moderate the violence of the local symptoms, and promote general functional harmony by propar depuratives. I speak here in general terms of those endemic forms of fever peculiar to a western and southern climate, and from the simple intermittent to its more malignant forms, and from the overpowering congestive, seen in the cold plague of the Mississippi, to the symptoms marking the yellow fever of the southern states. Of course the intelligent physician will modify or regulate his treatment according to the type the fever may assume during its progress, or the inflammatory action which may supervene. The fact of the intermittence or periodicity of all these forms of fever is well established; and the thought upon which I insist, with reference to their treatment, is, that in all forms of fever of a paroxysmal character, (and it is only of such that I speak,) the grand, out-standing, prominent idea to be cherished is, to prevent the repitition of paroxysms. In fact the early and judicious use of quinine, opium, capsicum, and other stimulants and sustenants of the brain and nervous system in our more malignant periodical fevers, before they become complicated with extensive local inflammation, is the very point on which the case is to turn for life or death. And the success of this treatment, in preference to the actual depletory system, is certainly very abundantly established. By it we immediately arrest the paroxysm, and with the arrest of the paroxysm, we generally have an improvement of the symptoms. The pulse, instead of being small, quick, and struggling, becomes fuller, softer, more distinct and natural; the skin assumes a more healthy condition, a free perspiration frequently forming a crisis to the fever; and when followed by proper alteratives, depuratives, tonics, and laxatives, the tongue improves in appearance, the secretions become restored, and the patient rapidly recovers. But if we wait, before adopting this practice, as recommended by a large majority of our most popular authors, until the secretions are restored, the pulse soft, and the febrile symptoms abated, we will wait until struggling nature, unaided, has overcome the disease; or until, in consequence of protracted congestions of organs whose functions are essential to life, inflammation shall have supervened which we may never be able to control.

